# Clinical outcomes of dose modification during pirfenidone treatment for IPF: A nationwide post-marketing surveillance study

**DOI:** 10.3389/fphar.2022.1025947

**Published:** 2023-01-10

**Authors:** Jieun Kang, Man Pyo Chung, Moo Suk Park, In Jae Oh, Heung Bum Lee, Young Whan Kim, Jong Sun Park, Soo Taek Uh, Yun Seong Kim, Yangjin Jegal, Jin Woo Song

**Affiliations:** ^1^ Division of Pulmonary and Critical Care Medicine, Department of Internal Medicine, Ilsan Paik Hospital, Goyang-si, South Korea; ^2^ Samsung Medical Center, Department of Pulmonary and Critical Care Medicine, Sungkyunkwan University School of Medicine, Seoul, South Korea; ^3^ Division of Pulmonary and Critical Care Medicine, Department of Internal Medicine, Severance Hospital, Yonsei University College of Medicine, Seoul, South Korea; ^4^ Department of Internal Medicine, Chonnam National University Medical School and Hwasun Hospital, Hwasun, South Korea; ^5^ Division of Pulmonary and Critical Care Medicine, Department of Internal Medicine, Research Center for Pulmonary Disorders, Jeonbuk National University Medical School and Hospital, Jeonju, South Korea; ^6^ Division of Respiratory-Allergy and Clinical Immunology, Department of Internal Medicine, Konkuk University Medical Center, Seoul, South Korea; ^7^ Division of Pulmonary and Critical Care Medicine, Department of Internal Medicine, Seoul National University Bundang Hospital, Seongnam, South Korea; ^8^ Division of Allergy and Respiratory Medicine, Department of Internal Medicine, Soonchunhyang University Seoul Hospital, Seoul, South Korea; ^9^ Department of Internal Medicine, Pusan National University Yangsan Hospital, Yangsan, South Korea; ^10^ Division of Pulmonary Medicine, Department of Internal Medicine, Ulsan University Hospital, Ulsan, South Korea; ^11^ Department of Pulmonary and Critical Care Medicine, Asan Medical Center, University of Ulsan College of Medicine, Seoul, South Korea

**Keywords:** idiopathic pulmonary fibrosis, pirfenidone, dose reduction, mortality, pulmonary function

## Abstract

**Background:** Pirfenidone, an antifibrotic medication approved for the treatment of idiopathic pulmonary fibrosis (IPF), often requires dose reduction owing to adverse events. In this study, we evaluated if pirfenidone’s reduced dose has any impact on clinical outcomes in patients with IPF.

**Methods:** We used the data of a prospective post-marketing study of pirfenidone conducted at 10 hospitals in South Korea from 2014 to 2017. Dose reduction was defined when the pirfenidone dose was temporarily or permanently reduced to manage adverse events or when the treatment dose failed to reach the standard dose. Study patients were classified based on the most frequently administered dose during 48-week follow-up—1800 mg, 1,200 mg, and <1,200 mg/days. The following clinical outcomes were compared between the groups: death, hospitalization, acute exacerbation, pulmonary function decline, and changes in severity of dyspnea and cough.

**Results:** The median follow-up duration in all 143 patients was 11 months. During the study period, 70.6% experienced at least one dose reduction. Patients treated with standard-dose pirfenidone tended to be young and had the lowest diffusing capacity. Pulmonary function changes did not differ depending on the pirfenidone dose. The three groups were not significantly different in terms of the proportion of death, hospitalization, and acute exacerbation. The symptom changes were also similar between the groups.

**Conclusion:** Reduced doses did not negatively impact clinical outcomes compared with the standard-dose pirfenidone in patients with IPF. Dose reduction may be a useful method to manage adverse events while maintaining therapeutic efficacy.

## Introduction

Idiopathic pulmonary fibrosis (IPF) is a chronic, progressive, fibrosing interstitial lung disease of unknown cause (Raghu et al., 2018). IPF is characterized by a poor prognosis with a median survival of 3–5 years after diagnosis without treatment (Ley et al., 2011; Nathan et al., 2011; Raghu et al., 2014). To date, only limited therapeutic options are available; pirfenidone or nintedanib (Maher and Strek, 2019). These antifibrotic medications are proven to attenuate the forced vital capacity’s (FVC) decline rate and have thus become the standard treatment for IPF (Noble et al., 2011; King et al., 2014; Richeldi et al., 2014).

Unfortunately, various adverse reactions may develop after antifibrotic treatment administration, which affects treatment adherence. According to a pooled analysis of safety data from five clinical trials of pirfenidone, almost all patients (97.6%) in the integrated population experienced one or more treatment-emergent adverse events (Lancaster et al., 2016). Pirfenidone’s common adverse events include gastrointestinal and dermatologic events such as nausea, diarrhea, rash, and photosensitivity (Noble et al., 2011; King et al., 2014; Lancaster et al., 2016). To manage adverse events and improve adherence, dose reductions and/or interruptions are frequently applied during pirfenidone treatment. A *posthoc* analysis of the pooled-phase 3 clinical trials of pirfenidone showed that 76.9% of patients in the pirfenidone group experienced at least one dose reduction, and 46.5% experienced at least one dose interruption (Nathan et al., 2018). In real-world studies, it has been also observed that a non-negligible proportion of patients are not tolerant to the full standard dose and thus take reduced doses (Ogura et al., 2015; Salih et al., 2016). A Japanese post-marketing surveillance (PMS) study found that the daily administered dose was generally ≤1,200 mg per day in approximately 62% of all patients (Ogura et al., 2015). Similarly, a nationwide Danish study showed that 15.9% of the participants discontinued treatment owing to adverse events, and 45.2% required dose adjustment to continue pirfenidone (Salih et al., 2016).

The recommended standard dose of pirfenidone approved in Asian countries is 1,800 mg/day whereas it is 2,403 mg/day in the US and Europe. As dose reduction is frequent in real clinical practice, a reduced dose may be given for a non-negligible period, the effects of which need to be assessed. This study aimed to evaluate whether pirfenidone treatment with a reduced dose impacts clinical outcomes of patients with IPF.

## Materials and methods

### Data source and study protocol

This study used data from a prospective PMS study of pirfenidone conducted at 10 hospitals in South Korea from 2014 to 2017. The PMS study was performed to obtain real-world data on safety, as well as investigate the treatment effects of pirfenidone in Korean patients with IPF. Data on the patients’ demographic information, concomitant medication, pulmonary function, respiratory symptoms (dyspnea and cough), and adverse events were obtained. Follow-up pulmonary function and symptom data were regularly collected at 12-week intervals. Spirometry was performed, and the diffusing capacity of the lungs for carbon monoxide (DL_CO_) was measured according to the American Thoracic Society (ATS)/European Respiratory Society (ERS) recommendations (Macintyre et al., 2005; Miller et al., 2005). Dyspnea was assessed based on the British Medical Research Council (MRC) grade (Bestall et al., 1999; Papiris et al., 2005), and the severity of cough was classified into four grades (none, mild, moderate, and severe) based on self-reported severity (Ogura et al., 2015). The follow-up duration was 48 weeks. The result of this PMS study has previously been published (Chung et al., 2020).

### Study patients

**FIGURE 1:**
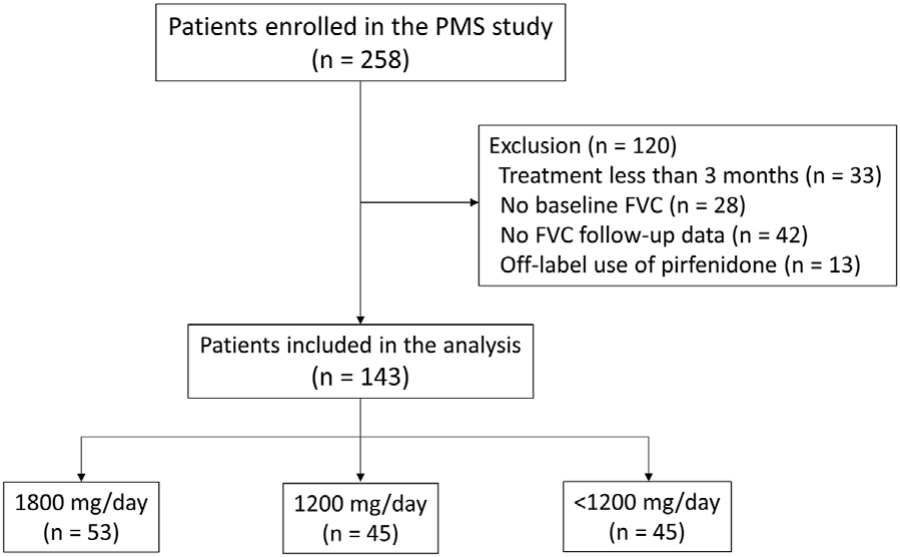
Study flowchart Abbreviation: PMS, post-marketing surveillance; FVC, forced vital capacity.

A total of 258 patients were enrolled in the PMS study. All patients were diagnosed with IPF at the host site based on the ATS/ERS/Japanese Respiratory Society/Latin American Thoracic Association guidelines (Raghu et al., 2011). Among them, patients who received pirfenidone for at least 3 months were included in the current study. Patients who received treatment for <3 months (*n* = 33), lacked FVC data at baseline (*n* = 28), lacked follow-up data on FVC (*n* = 42), or who received pirfenidone for off-label use (*n* = 13) were excluded, leaving 143 patients (Figure 1).

### Treatment and dose reduction

According to the package insert statement, patients started pirfenidone at 600 mg/day, and the daily dose was increased by 600 mg every 2 weeks until a total dose of 1,800 mg/day was reached. Based on patients’ tolerability and adverse events’ severity, the decision to increase or decrease the dose was made by each treating physician. In cases of severe adverse events, the drug could be temporarily discontinued based on the physician’s decision.

Dose reduction was defined when the pirfenidone dose was temporarily or permanently reduced to manage adverse events or when the treating dose failed to reach the standard dose owing to low tolerability. Dose reduction may result in some patients receiving doses lower than the standard dose for most of the treatment period. To determine the treatment effectiveness in those patients, we classified patients into the following three groups based on the most frequently taken dose during the follow-up period: 1,800, 1,200, and <1,200 mg/day groups.

### Study outcomes

Study outcomes included decline rates of FVC and DL_CO_, categorical changes in lung function, all-cause mortality, hospitalization (all-cause, respiratory-related, and non-respiratory-related), acute exacerbation (AE), changes in respiratory symptoms, and adverse events. AE was reported by attending physicians and was defined using the following criteria: Raghu et al., 2018 worsening dyspnea within 1 month (Ley et al., 2011); newly developed bilateral pulmonary infiltrates on computed tomography scan images; Raghu et al., 2014 a decrease of at least 10 mmHg in partial oxygen pressure compared with one at a stable state; Nathan et al., 2011 no identifiable precipitating factors such as infection or heart failure (Azuma et al., 2005; Taniguchi et al., 2010). Adverse events were defined using the preferred terms in the Medical Dictionary for Regulatory Activities version 21.0.

### Statistical analysis

Data are presented as number (percentage) and mean ± standard deviation for categorical variables and continuous variables, respectively. Student’s t-test was used to analyze continuous variables, whereas chi-square and Fisher’s exact tests were used to analyze categorical variables. The gender-age-physiology (GAP) index was calculated as Ley et al. (2012) proposed in 2012. The GAP stage was determined based on the total GAP index score: stage I (0–3 points), stage II (4–5 points), and stage III (6–8 points). The decline rates of lung function were calculated using a linear mixed model and compared between the different dose groups. Age, sex, smoking status, and baseline values of FVC and DL_CO_, were adjusted in the linear mixed model. Categorical changes in FVC and DL_CO_ were defined based on the absolute difference in the percent of the predicted values (% pred.) between baseline and end of the study; improvement was defined when the absolute change was +10% pred. or greater; worsening was defined when the absolute change was −10% pred. or greater and stable when neither of the criteria was met. The categorical changes in respiratory symptoms (dyspnea and cough) were classified as improved (decreased score), stable (no change), or worsened (increased score) by calculating the absolute changes in the British MRC grades and cough severity scores. Effects of concomitant medications on dose reduction were analyzed using logistic or linear regression.

## Results

### Baseline characteristics

The mean age of all patients was 67.5 years, and 73.4% were male. The mean FVC and DL_CO_ were 66.7% pred. and 53.4% pred., respectively. The median time from IPF diagnosis to pirfenidone treatment was 12 months (interquartile range [IQR] = 1–43 months). The median follow-up duration was 11 months (IQR = 9–12 months).

Baseline characteristics of study patients according to the most frequently received pirfenidone dose are illustrated in Table 1. In 90 patients (62.9%), the most frequently administered dose was less than the standard dose, with 45 patients each in the 1,200 mg and <1,200 mg groups. In the <1,200 mg group, most patients (95.6%) received 600 mg/day; there was one patient each who received 200 mg and 400 mg per day, respectively. Patients in the 1,800 mg group tended to be younger and had significantly lower DL_CO_ and shorter time from IPF diagnosis compared to those in other groups. Significantly more patients received N-acetylcysteine before pirfenidone in the <1,200 mg group whereas none received N-acetylcysteine in the 1,800 mg group. Prior treatment with corticosteroid was not significantly different between the three groups. There was a significant difference in the severity of respiratory symptoms between groups; patients in the 1,800 mg group included more patients with a mild degree of cough and MRC grade 1 dyspnea.

**TABLE 1:** Baseline characteristics of the study participants.

	1,800 mg	1,200 mg	<1,200 mg	*p*-value
Number of patients	53	45	45	
Age, years	65.7 ± 6.5	68.2 ± 8.7	68.9 ± 6.6	.079
Men	41 (77.4)	32 (71.1)	32 (71.1)	.716
Smoking				.246
Smoker	6 (11.3)	1 (2.2)	1 (2.2)	
Ex-smoker	32 (60.4)	28 (62.2)	29 (64.4)	
Non-smoker	15 (28.3)	16 (35.6)	15 (33.3)	
FVC, %pred	65.0 ± 14.0	65.8 ± 15.7	69.6 ± 14.3	.267
DL_CO_, %pred	48.9 ± 14.7	56.8 ± 19.6	55.6 ± 14.4	.038
Mean GAP index^a^	3.8 ± 1.4	3.7 ± 1.4	3.7 ± 1.2	.885
GAP stage^a^				.128
I	23 (43.4)	15 (36.6)	20 (45.5)	
II	23 (43.4)	23 (56.1)	24 (54.5)	
III	7 (13.2)	3 (7.3)	0 (0.0)	
Cough				<.001
Mild	30 (56.6)	10 (22.7)	5 (11.4)	
Moderate	20 (37.7)	26 (59.1)	19 (43.2)	
Severe	3 (5.7)	7 (15.9)	20 (45.5)	
Very severe	3 (5.7)	7 (15.9)	20 (45.5)	
Dyspnea (MRC scale)				.025
1	16 (30.2)	6 (13.6)	7 (15.6)	
2	24 (45.3)	29 (65.9)	26 (57.8)	
3	12 (22.6)	5 (11.4)	12 (26.7)	
4	1 (1.9)	4 (9.1)	0 (0.0)	
Comorbidity				
Hypertension	14 (26.4)	10 (22.2)	14 (31.1)	.634
Diabetes	12 (22.6)	11 (24.4)	12 (26.7)	.899
Chronic renal disease	0 (0.0)	2 (4.4)	0 (0.0)	.110
Chronic liver disease	2 (3.8)	3 (6.7)	5 (11.1)	.363
Cardiovascular disease	2 (3.8)	2 (4.4)	3 (6.7)	.792
COPD	1 (1.9)	2 (4.4)	1 (2.2)	.717
GERD	9 (17.0)	11 (24.4)	7 (15.6)	.507
Lung cancer, active	1 (1.8)	1 (2.2)	2 (4.4)	.717
Other malignancy, active	2 (3.8)	0 (0.0)	4 (8.9)	.108
Time from IPF diagnosis, m	16.7 ± 23.6	32.2 ± 34.8	27.6 ± 30.2	.030
Prior treatment				
N-acetylcysteine	0 (0.0)	4 (8.9)	7 (15.6)	.006
Corticosteroids	0 (0.0)	4 (8.9)	1 (2.2)	.593

Data are presented as the number (%) or mean ± standard deviation. FVC, forced vital capacity; DL_CO_, diffusing capacity of the lung for carbon monoxide; GAP, Gender-Age-Physiology; IPF, idiopathic pulmonary fibrosis; MRC, Medical Research Council; COPD, chronic obstructive pulmonary disease; GERD, gastroesophageal reflux disease; IPF, idiopathic pulmonary fibrosis.

^a^ The GAP index and GAP stage were assessed in 138 patients because five patients did not have baseline DL_CO_ data (53, 41, 44 patients in the 1,800, 1,200, and <1,200 mg group, respectively).

### Dose reduction and pirfenidone discontinuation

**FIGURE 2:**
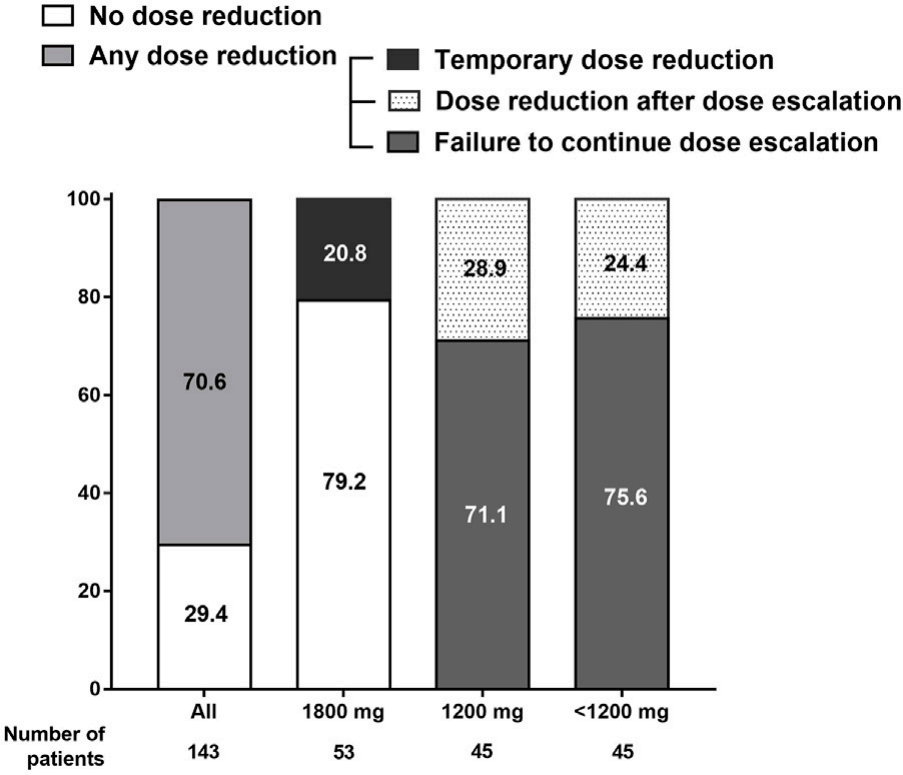
Adjustment of pirfenidone dose during the treatment course. Overall, 70.6% of the study participants experienced dose reduction at least once during the study period.

Figure 2 shows how the pirfenidone dose changed during the treatment course. Overall, 29.4% of all study patients received a pirfenidone dose according to the protocol and maintained the standard dose without dose reduction. The remaining 70.6% of the patients experienced at least one dose reduction during the study period.

In the 1,800 mg group, 79.2% of the patients maintained the standard dose during the follow-up; the remaining 20.8% experienced temporary dose reduction but were able to re-escalate to 1,800 mg. In the 1,200 mg and <1,200 mg groups, 71.1% and 75.6% of the patients failed to further increase the pirfenidone dose, respectively, owing to low tolerability at a dose lower than the standard dose; 28.9% and 24.4% attempted to increase the dose up to 1,800 and 1,200 mg/day, respectively, but returned to the reduced doses owing to adverse events.

Among the study patients, permanent discontinuation of pirfenidone occurred in 43 patients (30.1%) during the study period; 17, 12, and 14 patients discontinued pirfenidone in the 1,800, 1,200, and <1,200 mg groups, respectively. The most common cause of discontinuation was development of adverse events (60.5%); more patients stopped pirfenidone usage owing to adverse events in the <1,200 mg group (78.6%) than those in the 1,800 mg (52.9%) or 1,200 mg (50.0%) group, although the difference was not statistically significant (*p* = .238). Other reasons for discontinuation were patient desire (20.9%), loss to follow-up (11.6%), and lack of efficacy (7.0%).

### Effect of concomitant medications on dose reduction

Digestive medications were the most commonly administered drugs (48.6%) followed by proton pump inhibitors (34.7%) as shown in Supplementary Table S1. Any specific class of medications or polypharmacy (defined as medications ≥5) did not appear to affect dose reduction (Supplementary Table S1). Increasing number of medications was also not associated with dose reduction (*β* = 1.128, *p* = .144).

### Lung function decline

**FIGURE 3:**
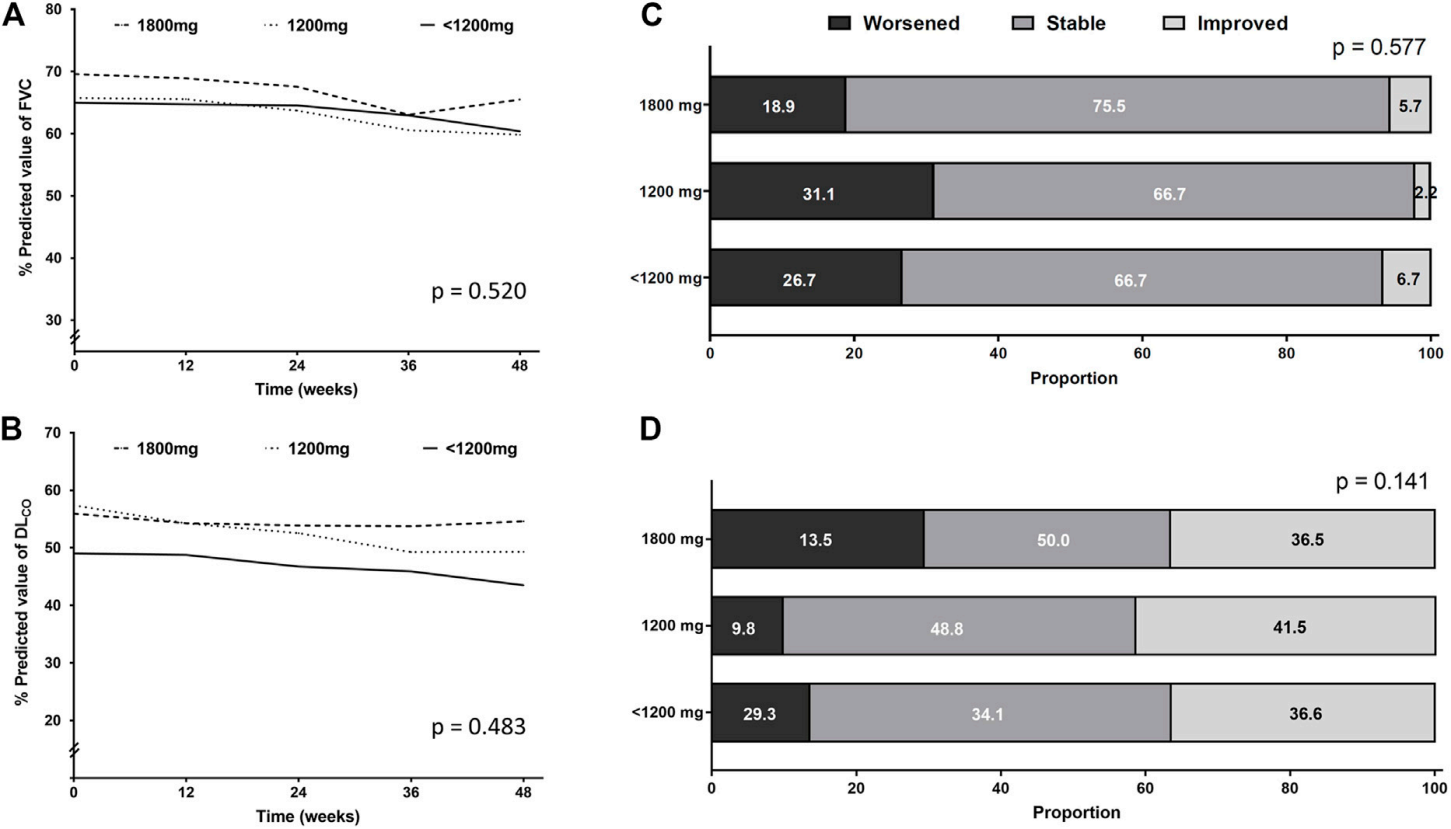
Pulmonary function changes according to the pirfenidone dose administered for the longest time. Longitudinal changes in **(A)** FVC and **(B)** DL_CO_. Categorical evaluation of changes in **(C)** FVC and **(D)** DL_CO_. Abbreviation: FVC, forced vital capacity; DL_CO_, diffusing capacity of the lung for carbon monoxide.

Figures 3A, B show the unadjusted mean changes of FVC and DL_CO_ according to the dose category, respectively. During the 48-week follow-up period, the three groups did not show significant differences in the decline rates of FVC and DL_CO_. The age-and sex-adjusted changes from the baseline values of FVC and DL_CO_ at 12, 24, 36, and 48 weeks are shown in Supplementary Tables S2, S3. At each time point, there was no significant difference between the three groups.

Lung function decline was also assessed categorically. The proportion of patients whose FVC or DL_CO_ worsened, improved, or stayed stable showed no significant difference between the three groups (Figures 3C, D).

### Mortality, hospitalization, and AE

Table 2 shows the number of all-cause deaths, hospitalizations, and AEs in the three groups. Overall, three deaths, 21 hospitalizations, and 4 AEs occurred during the study period. No significant difference was found in the proportion of patients who died, were hospitalized, or experienced AE between the different dose groups.

**TABLE 2:** Clinical outcomes according to the most frequently used pirfenidone dose.

	1,800 mg	1,200 mg	<1,200 mg	*p*-value
Number	53	45	45	
Follow-up duration (months)	11.0 [7.0, 12.0]	11.0 [10.0, 12.0]	11.0 [9.5, 11.0]	.523
Death	0 (0.0)	2 (4.4)	1 (2.2)	.310
Admission	7 (13.2)	7 (15.6)	7 (15.6)	.929
Respiratory	5 (71.4)	7 (100.0)	5 (71.4)	.635
Non-respiratory	2 (28.6)	0 (0.0)	2 (28.6)	.381
Acute exacerbation	1 (1.9)	2 (4.4)	1 (2.2)	.717

Data are presented as a median [interquartile range] or number (%).

### Changes in respiratory symptoms

**FIGURE 4:**
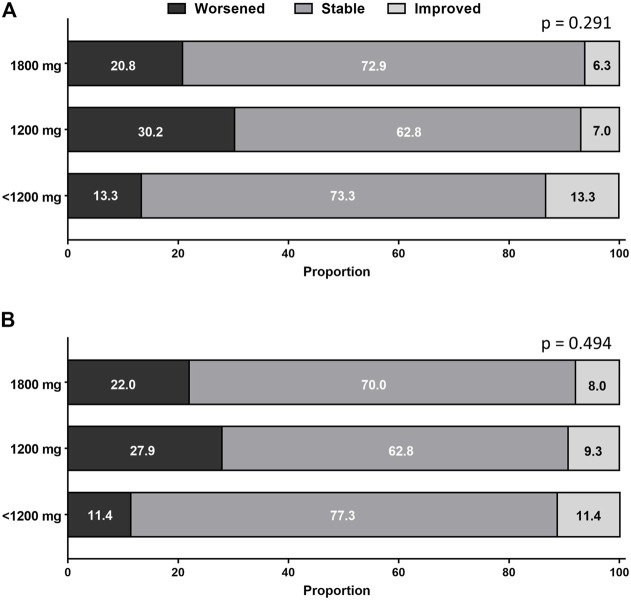
Categorical changes in **(A)** dyspnea and **(B)** cough according to pirfenidone dose. The proportion of patients who experienced improvement, no change, or worsening in respiratory symptoms did not significantly differ between the groups.

Categorical changes in respiratory symptoms according to the pirfenidone dose are shown in Figure 4. Dyspnea and cough severities remained stable in most patients during pirfenidone treatment. The proportion of patients who experienced improvement, no change, or worsening in dyspnea (Figure 4A) or cough (Figure 4B) was not significantly different between the groups.

### Adverse events

Supplementary Table S4 shows the frequency of adverse events. The most common adverse event was anorexia (32.2%), which tended to develop more frequently in patients in the 1,800 mg group (43.4%, 28.9%, and 22.2% in the 1,800 mg, 1,200 mg, and <1,200 mg group, respectively) than in the other groups. Photosensitivity was the second most common adverse event (16.1%), and it occurred more frequently in patients in the 1,800 mg group (30.2%, 13.3%, and 2.2% in the 1,800, 1,200, and <1,200 mg group, respectively) than in the other groups.

## Discussion

In this study, we found that 70.6% of the study patients experienced at least one dose reduction (temporary or permanent) during the 48-week study period. This dose reduction resulted in patients with the longest administered dose during the study period being lower than the standard dose. When patients were classified based on the most frequently administered dose, 62.9% of the study patients were either in the 1,200 mg/day or <1,200 mg group, whereas the rest were in the 1,800 mg/day group. In terms of treatment effectiveness, we did not find a significant difference in the clinical outcomes between the three groups.

Dose reduction or temporary dose interruption is an effective method to manage adverse events associated with pirfenidone (Costabel et al., 2014). One concern regarding this strategy is that the reduced pirfenidone dose may result in decreased effectiveness. According to our study, the clinical outcomes were not significantly different in patients who most frequently received a dose lower than the standard dose compared with those in the standard dose group. Our results are in line with previous studies (Nathan et al., 2018; Song et al., 2020). One retrospective single-center study in South Korea assessed the outcomes of 142 patients with IPF who were receiving pirfenidone treatment for >6 months (Song et al., 2020). The investigators classified patients into two groups according to the average dose the patients took during the treatment period—a low dose (<1,200 mg of pirfenidone per day) and high dose (≥1,200 mg per day). Similar to our study results, the groups showed no significant difference in the FVC decline rate during the treatment’s first year (−88.4 and −94.7 mL in the low-dose and high-dose groups, respectively). However, this result may be limited owing to the retrospective nature of the study. The strength of our study is that it used pulmonary function data, which were prospectively collected at regular intervals from multiple institutions. In addition, our study evaluated not only changes in FVC but also several aspects such as mortality, hospitalization, AE, and changes in respiratory symptoms.

Interestingly, some patients who received a dose of pirfenidone lower than the standard were revealed to have failed to continue dose escalation before reaching the standard dose. Pirfenidone was initiated at doses of 200 mg three times a day in accordance with the package insert statement, and the dose was increased based on patients’ tolerability and the severity of adverse events. Previous studies have shown that gastrointestinal-related adverse events appear mostly at the beginning of treatment and tend to decline over time (Valeyre et al., 2014; Lancaster et al., 2017); but given our finding that many patients failed to continue dose escalation throughout the study period, dose reduction may be necessary throughout the treatment period and not only at the beginning. A previous posthoc analysis by Nathan et al. also showed that dose reduction occurred throughout the year of treatment (Nathan et al., 2018). Therefore, our finding that dose reduction did not result in worse clinical outcomes has clinical significance.

This study has some limitations that should be addressed. First, the study duration of 48 weeks may have been too short to detect any significant difference in clinical outcomes such as mortality or AE. With a longer observation period, we might have observed more cases resulting in death or AE. In a previous study, patients receiving pirfenidone at a dose lower than 1,200 mg/day showed a similar AE rate to those receiving a higher dose in a median 2-year observation period, although the study did not show data on mortality (Song et al., 2020). Further studies with a long-term observation period may help clarify the long-term outcomes of dose reduction. Second, we did not use the definition of AE proposed in an international working group report by Collard et al., 2016. The current definition of AE does not require a decrease in partial oxygen pressure and does not mandate exclusion of causes (Collard et al., 2016). The definition used in this study is from clinical trials of pirfenidone conducted in Japan (Azuma et al., 2005; Taniguchi et al., 2010) and is stricter than the one suggested by Collard et al., which might have underestimated the AE rate. Lastly, the number of patients may have been too small to generalize the findings of our study. Previous real-world studies have shown that lower dose pirfenidone was not inferior in effectiveness (Song et al., 2020; Hwang et al., 2021), but further studies with a larger number of patients should be conducted to draw a firm conclusion.

In conclusion, patients treated with a reduced dose of pirfenidone showed similar clinical outcomes compared to those receiving the standard-dose pirfenidone. Dose reduction may be a useful method to manage adverse events while maintaining therapeutic efficacy.

## Data Availability

The datasets presented in this article are not readily available because of the issue related to individual patient confidentiality but are available from the corresponding author upon reasonable request with the permission of Ildong Pharmaceutical Co., Ltd. Requests to access the datasets should be directed to JWS, jwsongasan@gmail.com.
