# Supplementary material for: Clinical outcomes of dose modification during pirfenidone treatment for IPF: A nationwide post-marketing surveillance study
Source: Front Pharmacol. 2023 Jan 10;13:1025947. doi: 10.3389/fphar.2022.1025947 (PMC9871582; doi:10.3389/fphar.2022.1025947)
Supplement: Supplementary file 1 [file DataSheet1.docx]

**Clinical outcomes of dose modification during pirfenidone treatment for IPF: A nationwide post-marketing surveillance study**

Jieun Kang^1^, Man Pyo Chung^2^, Moo Suk Park^3^, In Jae Oh^4^, Heung Bum Lee^5^, Young Whan Kim^6^, Jong Sun Park^7^, Soo Taek Uh^8^, Yun Seong Kim^9^, Yangjin Jegal^10^, Jin Woo Song^11^

^1^Division of Pulmonary and Critical Care Medicine, Department of Internal Medicine, Ilsan Paik Hospital, Goyang, South Korea

^2^Department of Pulmonary and Critical Care Medicine, Samsung Medical Center, Sungkyunkwan University School of Medicine, Seoul, South Korea

^3^Division of Pulmonary and Critical Care Medicine, Department of Internal Medicine, Yonsei University College of Medicine, Severance Hospital, Seoul, South Korea

^4^Department of Internal Medicine, Chonnam National University Medical School and Hwasun Hospital, Hwasun, South Korea

^5^Division of Pulmonary and Critical Care Medicine, Department of Internal Medicine, Research Center for Pulmonary Disorders, Jeonbuk National University Medical School and Hospital, Jeonju, South Korea

^6^Division of Respiratory-Allergy & Clinical Immunology, Department of Internal Medicine, Konkuk University Medical Center, Seoul, South Korea

^7^Division of Pulmonary and Critical Care Medicine, Department of Internal Medicine, Seoul National University Bundang Hospital, Seongnam, South Korea

^8^Division of Allergy and Respiratory Medicine, Department of Internal Medicine, Soonchunhyang University Seoul Hospital, Seoul, South Korea

^9^Department of Internal Medicine, Pusan National University Yangsan Hospital, Yangsan, South Korea

^10^Division of Pulmonary Medicine, Department of Internal Medicine, Ulsan University Hospital, Ulsan, South Korea

^11^Department of Pulmonary and Critical Care Medicine, Asan Medical Center, University of Ulsan College of Medicine, Seoul, South Korea

Supplementary Table S1. Effect of specific class of medication on dose reduction

| Class | Number (%) | Odds ratio (95% CI) | p-value |
| --- | --- | --- | --- |
| Digestive medications | 103 (48.6) | 1.189 (0.562–2.517) | 0.651 |
| Proton pump inhibitors | 102 (34.7) | 0.524 (0.236–1.160) | 0.111 |
| Antihistamine | 85 (59.4) | 1.542 (0.774–3.072) | 0.218 |
| H2 blockers | 27 (18.9) | 1.001 (0.421–2.384) | 0.998 |
| Anti-hypertensive agents | 20 (6.8) | 0.865 (0.329–2.276) | 0.769 |
| Anti-diabetic agents | 12 (4.1) | 0.810 (0.244–2.692) | 0.730 |
| Cardiovascular medications | 5 (1.7) | 0.379 (0.061–2.344) | 0.296 |
| Polypharmacy (≥5 medications) | 39 (27.3) | 1.250 (0.576–2.711) | 0.572 |

CI, confidence interval.

Supplementary Table S2. Mean changes in FVC from the baseline value (% of the predicted value)

|  | 12 weeks | | | 24 weeks | | | 36 weeks | | | 48 weeks | | |
| --- | --- | --- | --- | --- | --- | --- | --- | --- | --- | --- | --- | --- |
|  | LS means | SE | p-value | LS means | SE | p-value | LS means | SE | p-value | LS means | SE | p-value |
| 1800mg | 0.7 | 2.0 | 0.958 | -1.1 | 1.9 | 0.747 | -6.4 | 2.1 | 0.050 | -2.8 | 2.1 | 0.805 |
| 1200mg | 0.6 | 1.8 |  | -0.9 | 1.8 |  | -3.4 | 1.9 |  | -4.2 | 1.8 |  |
| <1200mg | 0.1 | 1.6 |  | 0.3 | 1.5 |  | -0.8 | 1.6 |  | -3.1 | 1.6 |  |

LS means, least squared means; SE, standard error; FVC, forced vital capacity

Supplementary Table S3. Mean changes of DL_CO_ from the baseline value (% of the predicted value)

|  | 12 weeks | | | 24 weeks | | | 36 weeks | | | 48 weeks | | |
| --- | --- | --- | --- | --- | --- | --- | --- | --- | --- | --- | --- | --- |
|  | LS means | SE | p-value | LS means | SE | p-value | LS means | SE | p-value | LS means | SE | p-value |
| 1800mg | 1.0 | 2.7 | 0.755 | 1.6 | 2.5 | 0.195 | 0.0 | 2.9 | 0.190 | 2.0 | 3.1 | 0.053 |
| 1200mg | 0.3 | 2.5 |  | -3.6 | 2.5 |  | -6.4 | 2.7 |  | -6.2 | 2.5 |  |
| <1200mg | -1.2 | 2.2 |  | -2.7 | 2.0 |  | -3.3 | 2.2 |  | -5.3 | 2.1 |  |

LS means, least squared means; SE, standard error; DL_CO_, diffusing capacity of the lungs for carbon monoxide

Supplementary Table S4. Adverse events

|  | 1800 mg | 1200 mg | <1200 mg | p-value |
| --- | --- | --- | --- | --- |
| Number of patients | 53 | 45 | 45 |  |
| Anorexia | 23 (43.4) | 13 (28.9) | 10 (22.2) | 0.070 |
| Nausea | 9 (17.0) | 6 (13.3) | 4 (8.9) | 0.501 |
| Epigastric discomfort | 7 (13.2) | 9 (20.0) | 5 (11.1) | 0.457 |
| Weight loss | 2 (3.8) | 0 (0.0) | 2 (4.4) | 0.381 |
| Dyspepsia | 1 (1.9) | 0 (0.0) | 2 (4.4) | 0.336 |
| Constipation | 1 (1.9) | 3 (6.7) | 2 (4.4) | 0.498 |
| Diarrhea | 0 (0.0) | 0 (0.0) | 1 (2.2) | 0.334 |
| Photosensitivity | 16 (30.2) | 6 (13.3) | 1 (2.2) | 0.001 |
| Pruritus | 7 (13.2) | 6 (13.3) | 5 (11.1) | 0.937 |
| Liver enzyme elevation | 5 (9.4) | 2 (4.4) | 0 (0.0) | 0.096 |
| General weakness | 2 (3.8) | 1 (2.2) | 1 (2.2) | 0.863 |

Data are presented as the number (%).
